# Cadaveric analyses of injectate distribution patterns in ultrasound-guided rotator interval, dual-target, and posterior glenohumeral injections

**DOI:** 10.1186/s13244-026-02255-y

**Published:** 2026-03-18

**Authors:** Ondřej Naňka, Kamal Mezian, Ke-Vin Chang, Wei-Ting Wu, Vincenzo Ricci, Levent Özçakar

**Affiliations:** 1 Institute of Anatomy, Charles University, First Faculty of Medicine, Prague, Czech Republic; 2 Department of Rehabilitation Medicine, Charles University, First Faculty of Medicine and General University Hospital in Prague, Prague, Czech Republic; 3 Department of Physical Medicine and Rehabilitation, National Taiwan University Hospital, Bei-Hu Branch, Taipei, Taiwan; 4 Department of Physical Medicine and Rehabilitation, National Taiwan University College of Medicine, Taipei, Taiwan; 5 Center for Regional Anesthesia and Pain Medicine, Wang-Fang Hospital, Taipei Medical University, Taipei, Taiwan; 6 Physical and Rehabilitation Medicine Unit, Luigi Sacco University Hospital, ASST Fatebenefratelli-Sacco, Milano, Italy; 7 Department of Physical and Rehabilitation Medicine, Hacettepe University Medical School, Ankara, Turkey

**Keywords:** Shoulder, Rotator cuff, Pain, Ultrasonography, Intervention

## Abstract

**Objectives:**

Intra-articular corticosteroid injection is a standard treatment for adhesive capsulitis and is commonly performed via the rotator interval or posterior glenohumeral approaches, with prior evidence favoring the former. Injectate distribution varies by technique. Additionally, a dual-target approach combining rotator interval and subdeltoid bursal injections has been proposed for subacromial impingement. This study compared dye distribution among rotator interval, dual-target, and posterior glenohumeral injection techniques.

**Materials and methods:**

This cadaveric study evaluated dye spread in 18 shoulders from nine embalmed cadavers. Three ultrasound-guided techniques were assessed: rotator interval injection (15 mL), dual-target injection (10 mL rotator interval + 5 mL subdeltoid bursa), and posterior glenohumeral injection (15 mL). Following the injection, systematic dissection was performed. Staining of the subdeltoid bursa, long head of the biceps tendon sheath, anterior glenohumeral capsule, and posterior glenohumeral capsule was graded as absent, partial, or extensive.

**Results:**

Extensive staining of the biceps tendon sheath and anterior glenohumeral capsule was observed in all shoulders receiving rotator interval or dual-target injections, whereas posterior glenohumeral injections showed inconsistent coverage of these structures. Reliable subdeltoid bursal infiltration occurred only in the dual-target group (5/6 shoulders). The posterior glenohumeral capsule was most consistently and extensively stained following posterior glenohumeral injection (6/6), with moderate coverage in the rotator interval and dual-target groups. Infraspinatus infiltration was uncommon and observed only after posterior glenohumeral injection.

**Conclusion:**

The injection technique markedly influences shoulder injectate distribution. Rotator interval and dual-target approaches preferentially address anterior structures, the dual-target technique ensures subdeltoid bursal coverage, and the posterior approach most consistently infiltrates the posterior glenohumeral capsule. Technique selection should be guided by the predominant pathological target in adhesive capsulitis and related disorders.

**Critical relevance statement:**

Dual-target and rotator interval approaches reliably infiltrate the anterior capsule, making them suitable for adhesive capsulitis with biceps long head pathology, whereas the posterior glenohumeral approach primarily covers the posterior capsule and is less suitable for concomitant anterior shoulder disorders.

**Key Points:**

- The injection technique determines the injectate distribution in the shoulder.
- Dual-target and rotator interval injections ensure anterior capsule infiltration.
- The posterior approach best targets the posterior capsule.

**Graphical Abstract:**

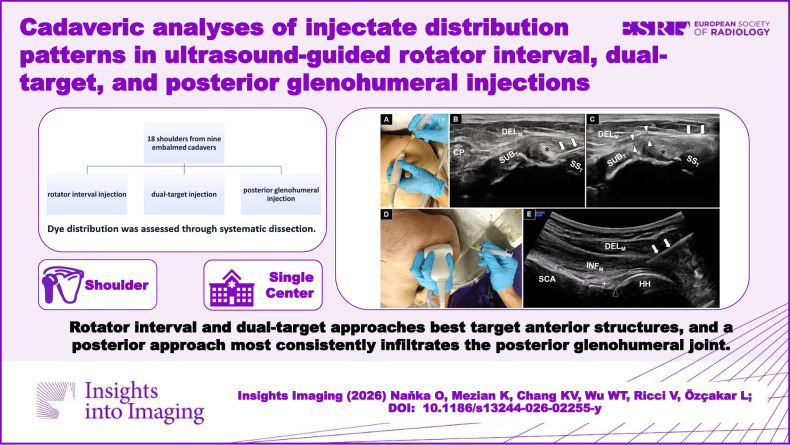

## Introduction

Adhesive capsulitis, commonly referred to as frozen shoulder, is a debilitating disorder characterized by progressive limitation of both active and passive glenohumeral motions accompanied by pain. Its prevalence is estimated at 2–5% in the general population [1] and is notably higher among individuals with metabolic comorbidities such as diabetes mellitus and thyroid disease [2]. Although conservative management, including analgesics, physical therapy, and activity modification, leads to symptomatic improvement in many cases, a considerable subset of patients remains refractory. For those individuals, intra-articular corticosteroid injection has been widely adopted as an evidence-based second-line intervention [3], with demonstrated efficacy in reducing pain and improving range of motion.

Among the commonly used techniques, the rotator interval and posterior glenohumeral injections are most frequently performed. Recent randomized controlled trials and meta-analyses have suggested that the rotator interval approach provides greater improvements in pain and motion compared to the posterior approach [4, 5]. Nevertheless, even though the glenohumeral capsule is anatomically continuous, injectate distribution may not be uniform, particularly when limited volumes are used. As such, there remain concerns about whether technique-dependent variations affect the clinical outcome.

Anatomically, the rotator interval is bordered by the supraspinatus and subscapularis tendons, with the coracoid process forming its base [6, 7]. It contains the biceps long head tendon, enveloped by the coracohumeral and superior glenohumeral ligaments. This area is a key site of pathology in adhesive capsulitis, where fibrosis contributes to loss of external rotation. By contrast, the posterior glenohumeral capsule, located beneath the infraspinatus and teres minor, provides an accessible sonographic window but may not reliably cover anterior structures. Since subdeltoid bursa pathologies may coexist with adhesive capsulitis, there is a rationale for combined or “dual-target” injections [8]. The latter technique deposits corticosteroid both into the rotator interval and the subdeltoid bursa. It has been shown in randomized controlled trials to reduce pain recurrence and prolong symptom relief, compared with standardized subacromial injections [9–11]. However, its specific role in adhesive capsulitis remains unclear. Although the rotator interval is targeted, the intra-articular volume is reduced compared with a pure rotator interval injection, raising questions about whether dye or medication adequately reaches the posterior capsule.

Injection volume itself may also influence the clinical outcome while managing adhesive capsulitis. A 2017 meta-analysis [12] reported that hydrodilatation with larger volumes (10–90 mL) facilitated faster recovery of motion than conventional injections, suggesting a possible mechanical effect in addition to pharmacological benefit.

This cadaveric study was therefore designed to compare the dye distribution patterns of three techniques: rotator interval, dual-target, and posterior glenohumeral injections. We aimed to determine whether key structures—the long head of the biceps tendon sheath, anterior glenohumeral joint, posterior glenohumeral joint, and subdeltoid bursa—are consistently infiltrated, and to identify any additional sites of dye spread. We considered that these findings may clarify anatomic patterns of injectate distribution and provide practical guidance for optimizing the injection technique in adhesive capsulitis and related shoulder conditions.

## Materials and methods

### Cadaveric specimens

This study was performed on nine cadavers donated to the Institute of Anatomy, First Faculty of Medicine at Charles University, Prague, Czech Republic. Ethical approval was granted by the Anatomical Donation Department, Charles University, Prague, authorizing the use of donated cadavers for scientific investigation and publication (see Supplementary Document 1: Documentation of Institutional Review Board Waiver for “Cadaveric Analyses of Injectate Distribution Patterns in Ultrasound-Guided Rotator Interval, Dual-Target, and Posterior Glenohumeral Injections”). As no living participants were involved, institutional review board approval was waived by Charles University. All cadavers had been preserved using the Fix-for-Life embalming method, which maintains soft tissue consistency, pliability, and coloration closely resembling that of living anatomy [13]. This preservation technique enabled injection procedures and subsequent dissections to be conducted under conditions that closely simulated the in vivo environment.

### Ultrasound equipment and injection techniques

All injections were performed using a Samsung HS30 ultrasound system (Samsung Medison Co., Ltd.). A high-frequency linear transducer (LN5–12) was applied to visualize superficial structures, including the rotator interval and subdeltoid bursa. A curvilinear transducer (C2–5) was utilized for deeper targets, such as the posterior glenohumeral joint. All procedures were performed by a physician (K.V.C.) with over 10 years of experience in musculoskeletal ultrasound-guided interventions, thereby ensuring methodological consistency and minimizing operator-dependent variability.

**Fig. 1:**
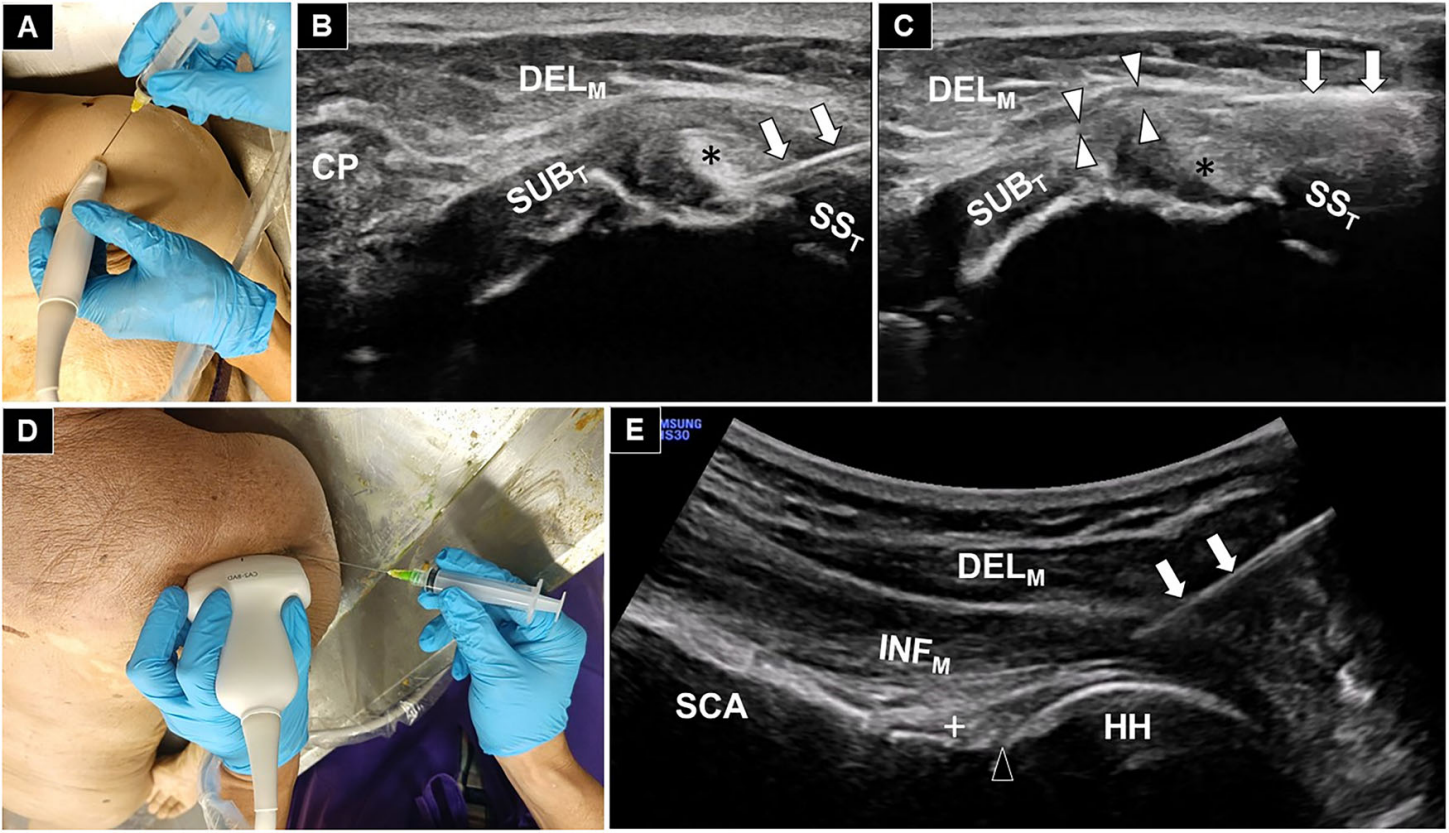
Transducer position and needle placement for ultrasound-guided rotator interval and dual-target injections (**A**), with corresponding ultrasound images showing injection into the peritendinous space of the long head of the biceps tendon (asterisk) (**B**) and the subdeltoid bursa (white arrowheads) (**C**). Transducer position and needle placement for the posterior glenohumeral joint injection (**D**) and corresponding ultrasound-guided injection into the posterior glenohumeral joint (black arrowhead) are shown in **E**. White arrows: needle trajectory; white cross: labrum. CP, coracoid process; DEL_M_, deltoid muscle; SS_T_, supraspinatus tendon; SUB_T_, subscapularis tendon; INF_M_, infraspinatus muscle; HH, humeral head; SCA, scapula

Three injection techniques were evaluated using 18 shoulders from nine cadavers, with each technique being applied to six shoulders. For the rotator interval injection, each cadaver was positioned supine (Fig. 1A). A linear transducer was positioned over the rotator interval to visualize the long head of the biceps tendon, along with the subscapularis and supraspinatus tendons and the overlying coracohumeral ligament. Under ultrasound guidance, 15 mL of green dye was injected into the peritendinous space surrounding the biceps tendon (Fig. 1B).

For the dual-target injection, cadavers were also positioned supine, and the same initial approach was adopted. After 10 mL of green dye was injected around the long head of the biceps tendon, the transducer was slightly repositioned to target the subdeltoid bursa, where an additional 5 mL was delivered (Fig. 1C).

For the posterior glenohumeral joint injection, the cadaver was positioned prone (Fig. 1D). A curvilinear transducer was aligned along the scapular spine and moved distally to identify the posterior glenohumeral joint. A 20-gauge, 7-cm needle was advanced in a lateral-to-medial trajectory to inject 15 mL of green dye solution into the posterior joint space (Fig. 1E).

### Dissection methods for dye distribution analysis

**Fig. 2:**
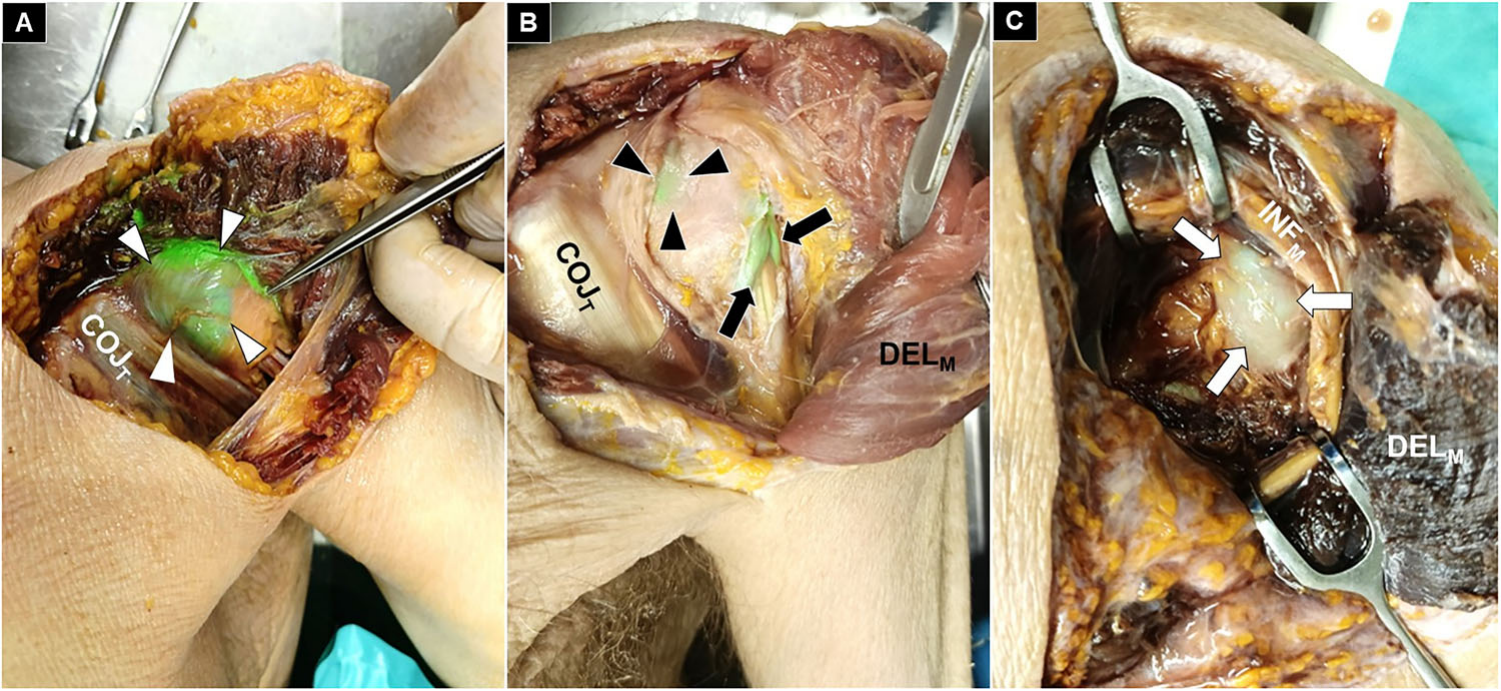
The subdeltoid bursa (white arrowheads) (**A**), biceps tendon sheath (black arrows) and anterior glenohumeral capsule (black arrowheads) (**B**), and posterior glenohumeral capsule (white arrows) (**C**) were examined for staining, with each structure incised only after confirming external dye presence. COJ_T_, conjoint tendon; DEL_M_, deltoid muscle; INF_M_, infraspinatus muscle

After injection, dye distribution was assessed through systematic dissection by an anatomist with over 10 years of cadaveric experience. For the anterior shoulder approach, an oblique incision was made along the interval between the anterior deltoid and pectoralis major, following the cephalic vein. After separating the muscle plane, retractors exposed the conjoined tendon of the short head of the biceps brachii, coracobrachialis, and pectoralis minor. The subdeltoid bursa (Fig. 2A), biceps tendon sheath (Fig. 2B) and the anterior glenohumeral capsule (Fig. 2B) were examined for staining. Each structure was incised only after confirming external dye presence to prevent leakage and ensure accurate mapping of intra-articular and bursal distributions.

For the posterior shoulder approach, an oblique incision was made along the interval between the deltoid and trapezius muscles. Dissection of this interval exposed the infraspinatus and teres minor muscles. The space in between was then opened, and the infraspinatus was incised when necessary to fully visualize the posterior glenohumeral capsule (Fig. 2C). As with the anterior approach, the capsule was initially inspected for dye infiltration prior to incision, to provide an unaltered assessment of the distribution pattern.

Regarding dye spread, assessments were performed collaboratively by two observers: one author (Kamal Mezian), who was not involved in the injection procedure, and the anatomist (Ondřej Naňka), who conducted the dissection. In cases of discrepant initial assessments, the anatomist made the final verification of dye distribution.

### Outcome assessment

**Fig. 3:**
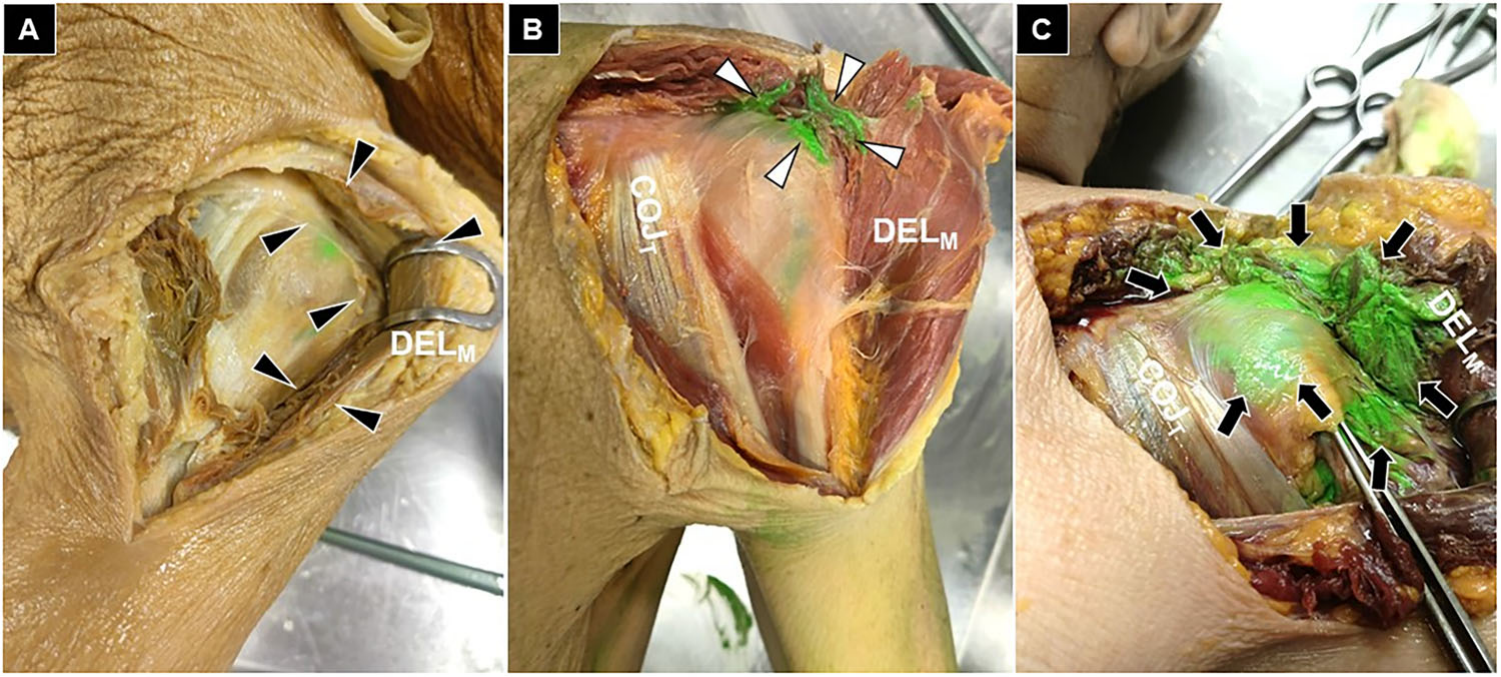
Cadaveric demonstration of absent (black arrowheads) (**A**), partial (white arrowheads) (**B**), and extensive (black arrows) (**C**) staining of the subdeltoid bursa. COJ_T_, conjoint tendon; DEL_M_, deltoid muscle

**Fig. 4:**
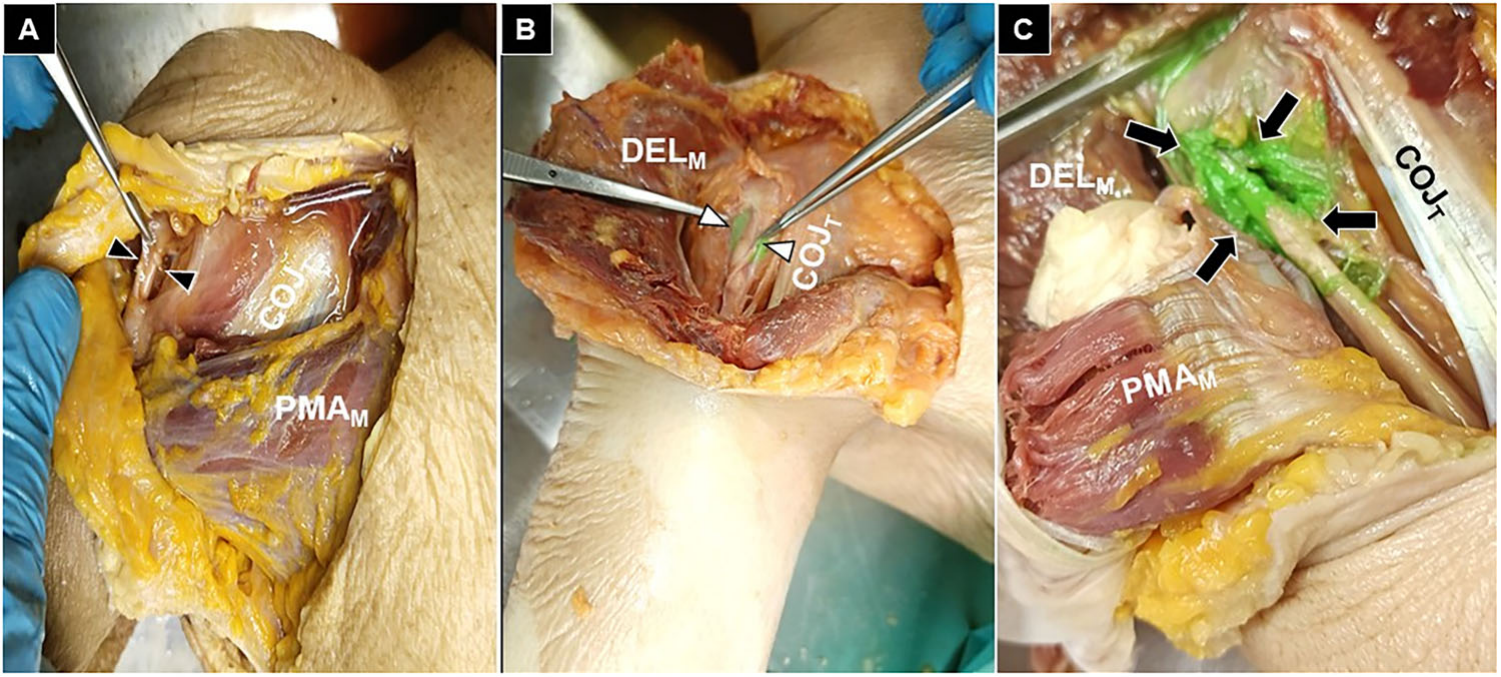
Cadaveric demonstration of absent (black arrowheads) (**A**), partial (white arrowheads) (**B**), and extensive (black arrows) (**C**) staining of the long head of the biceps tendon sheath. COJ_T_, conjoint tendon; PMA_M_, pectoralis major muscle; DEL_M_, deltoid muscle

**Fig. 5:**
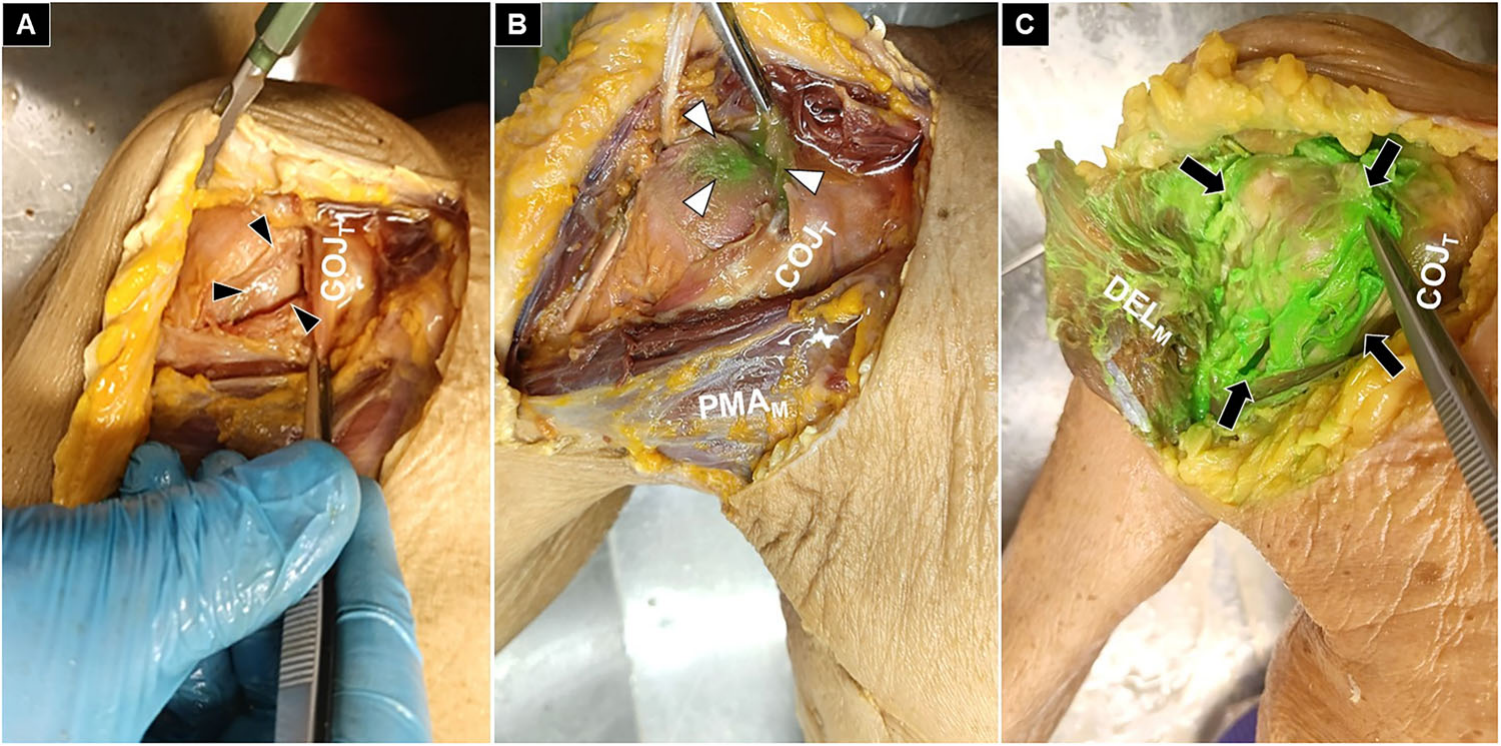
Cadaveric demonstration of absent (black arrowheads) (**A**), partial (white arrowheads) (**B**), and extensive (black arrows) (**C**) staining of the anterior glenohumeral joint. COJ_T_, conjoint tendon; PMA_M_, pectoralis major muscle; DEL_M_, deltoid muscle

**Fig. 6:**
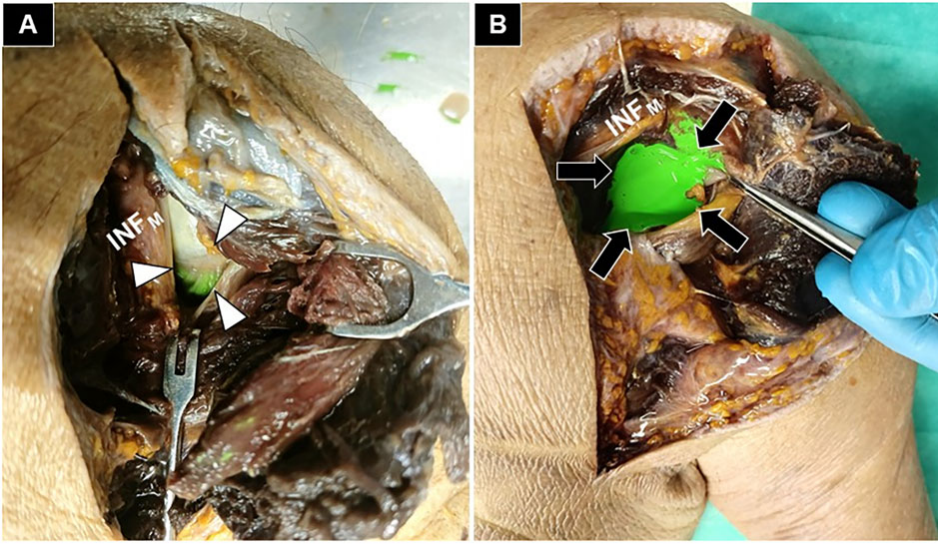
Cadaveric demonstration of partial (white arrowheads) (**A**) and extensive (black arrows) (**B**) staining of the posterior glenohumeral joint. None of the three techniques showed a complete absence of staining in this region. DEL_M_, deltoid muscle; INF_M_, infraspinatus muscle.

Each dissected structure was inspected, and the extent of dye infiltration was graded as absent (no staining), partial (< 50% of the structure stained), or extensive (≥ 50% of the structure stained). The primary outcome was the presence and distribution of green dye within predefined anatomical targets: the subdeltoid bursa (Fig. 3), the long head of the biceps tendon (Fig. 4), the anterior glenohumeral joint (Fig. 5), and the posterior glenohumeral joint (Fig. 6). This grading system provided a semi-quantitative assessment of dye dispersion among the different injection techniques.

### Statistical analysis

All statistical analyses were performed using IBM SPSS Statistics for Windows, version 21.0 (IBM Corp.). Staining grades were treated as categorical variables and were compared between groups using Fisher’s exact test. Continuous variables were analyzed with the Mann–Whitney U test. A *p*-value < 0.05 was considered statistically significant.

## Results

### Cadaver demographics

Nine cadavers (five females, four males) were included in this study. The overall mean body mass index was 23.0 ± 2.9 (range, 19–27), with no significant difference between sexes (female: 23.0 ± 2.6, male: 23.0 ± 3.7, *p* = 1.0).

### Dye distribution: descriptive findings

The dye distribution patterns are summarized in Table 1. The biceps tendon sheath demonstrated extensive staining in all six shoulders receiving rotator interval and dual-target injections. In the posterior glenohumeral injection group, three of six shoulders showed extensive staining, one showed partial staining, and two showed no staining.

**Table 1:** Distribution of staining grades across injection techniques

Structure	Technique	Absent (*n*)	Partial (*n*)	Extensive (*n*)
Biceps tendon sheath	Rotator interval injection	0	0	6
	Dual-target injection	0	0	6
	Posterior glenohumeral joint injection	2	1	3
Anterior glenohumeral joint	Rotator interval injection	0	0	6
	Dual-target injection	0	0	6
	Posterior glenohumeral joint injection	1	1	4
Subdeltoid bursa	Rotator interval injection	5	1	0
	Dual-target injection	1	0	5
	Posterior glenohumeral joint injection	6	0	0
Posterior glenohumeral joint	Rotator interval injection	0	1	5
	Dual-target injection	0	4	2
	Posterior glenohumeral joint injection	0	0	6
Infraspinatus muscle/fossa	Rotator interval injection	6	0	0
	Dual-target injection	6	0	0
	Posterior glenohumeral joint injection	4	2	0

Numbers indicate the count of shoulders (*n* = 6 per technique), demonstrating absent, partial, or extensive staining

For the anterior glenohumeral joint, all six shoulders in both the rotator interval and dual-target groups exhibited extensive staining. In contrast, the posterior glenohumeral group showed extensive staining in four shoulders, partial staining in one, and no staining in one.

Concerning differences in the subdeltoid bursa, extensive staining was observed in five of six shoulders following the dual-target injection. The remaining specimen exhibited no staining, with dye confined to the connective tissue contiguous with the superficial portion of the lateral bundle of the coracohumeral ligament (Supplementary Fig. 1A). In the rotator interval group, five shoulders showed no staining, and one demonstrated partial staining. In contrast, no staining was detected in any of the six shoulders in the posterior glenohumeral injection group.

The posterior glenohumeral joint exhibited the most consistent infiltration with the posterior glenohumeral injection, producing extensive staining in all six shoulders. The rotator interval group yielded extensive staining in five shoulders and partial staining in one. The dual-target group resulted in extensive staining in two shoulders and partial staining in four. Importantly, none of the techniques demonstrated truly absent staining in this region.

Overall, staining in the infraspinatus muscle/fossa was infrequent. Two of six shoulders in the posterior glenohumeral group showed partial infiltration (Supplementary Fig. 1B), while all shoulders in the rotator interval and dual-target groups showed no staining.

### Dye distribution: statistical findings

Fisher’s exact test corroborated the observed descriptive trends; however, differences among injection techniques for the biceps long head tendon (*p* = 0.126), anterior glenohumeral joint capsule (*p* = 0.343), and infraspinatus muscle (*p* = 0.105) did not reach statistical significance. In contrast, a significant difference was identified in the subdeltoid bursa (*p* = 0.004), where the dual-target injection yielded the most consistent and extensive staining. The posterior glenohumeral joint also exhibited significant variability (*p* = 0.027), with the posterior glenohumeral injection achieving the most reliable and extensive dye infiltration.

## Discussion

This study served as a pilot investigation to evaluate the anterior spread of injectate following posterior glenohumeral injection—an aspect rarely addressed in cadaveric research. Previous studies have demonstrated that the long head of the biceps tendon sheath communicates with the glenohumeral joint [14, 15]; however, few have examined whether injectate introduced posteriorly can traverse anteriorly across the joint space. The present work also compared this posterior trajectory with anteriorly directed approaches through the rotator interval.

### Dye distribution and anatomical interpretation

Dye distribution varied according to the injection technique. The biceps tendon sheath was extensively stained in all shoulders receiving rotator interval and dual-target injections, whereas the posterior glenohumeral approach produced extensive staining in three shoulders (partial in one and none in two). This discrepancy likely reflects anatomical factors. The rotator interval and dual-target techniques positioned the needle near the peritendinous portion of the long head of the biceps tendon, facilitating direct infiltration. Conversely, in the posterior approach, the dye had to traverse from the posterior capsule toward the anterior capsule and rotator interval. Along this route, the coracohumeral and superior glenohumeral ligaments likely acted as partial barriers [16], limiting anterior diffusion and contributing to inter-specimen variability. Although prior cadaveric studies have described the communication between the biceps tendon sheath and the glenohumeral joint, few have investigated the anterior propagation of the injectate following posterior injection. Gofeld et al [15] demonstrated that ultrasound-guided biceps tendon sheath injections produced intra-articular dye spread in 11 of 12 specimens, confirming anatomical continuity between the sheath and the joint capsule. The present study extends this understanding by illustrating the reverse pathway, showing that injectate introduced posteriorly can diffuse anteriorly across the joint space.

The anterior glenohumeral joint showed the most uniform infiltration. All six shoulders in the rotator interval and dual-target groups exhibited extensive staining, consistent with the anatomical continuity between the biceps tendon sheath and the anterior glenohumeral capsule [14, 17]. With injection volumes of 10–15 mL, the dye readily dispersed into this region. In the posterior glenohumeral group, four shoulders demonstrated extensive staining, one partial, and one none. This reduced consistency likely reflected the longer diffusion path around the humeral head. Nevertheless, the anterior glenohumeral capsule was more consistently infiltrated than the biceps tendon sheath following posterior injection, as it represented a closer and less anatomically constrained target.

The subdeltoid bursa was consistently stained only in the dual-target group, where five of six shoulders exhibited extensive dye infiltration. This finding corresponded with the intended injection route, as 5 mL of dye was directly delivered into the bursa following intra-articular injection. The remaining specimen in this group showed no bursal staining, with dye confined to connective tissue contiguous with the superficial fibers of the coracohumeral ligament. This observation supports the anatomical continuity between the subdeltoid subacromial bursae and the coracohumeral ligament within the rotator interval, as described by Kennedy et al [8], who noted that the bursal roof attaches to the deep surface of the acromion and coracoacromial ligament, whereas its floor fuses with the supraspinatus tendon and greater tubercle. In contrast, the rotator interval group showed only one case of partial staining—likely due to backflow during needle passage—while the posterior group showed none, as this approach does not communicate with the bursal compartment.

The posterior glenohumeral joint was most consistently infiltrated by the posterior glenohumeral injection, with all six shoulders showing extensive staining. The rotator interval approach achieved comparable coverage in five shoulders, with one showing partial staining, whereas the dual-target group demonstrated extensive staining in two shoulders and partial in four. This difference can be attributed to the injectate distribution: in the rotator interval approach, the entire 15 mL was delivered intra-articularly, facilitating posterior spread, while in the dual-target technique, only 10 mL entered the joint and 5 mL was diverted into the bursa. These results parallel the findings of Nwawka et al [18], who reported that injectate flow between the biceps tendon sheath and joint occurred consistently with moderate-to-large volumes (5–10 mL) but inconsistently with smaller volumes (2 mL), underscoring the influence of injection volume on intra-articular spread.

Because the infraspinatus muscle and fossa lack direct communication with the glenohumeral capsule, infiltration in this region was observed only in the posterior glenohumeral group (two of six shoulders). This finding is anatomically explicable, as injection under pressure may have led to extravasation along the needle track or through capsular margins. In contrast, the anterior approaches—being anatomically distant from the infraspinatus region—showed no staining.

Furthermore, the selection of a 15-mL volume for hydrodilatation was based on the volume we routinely use for single-shot capsular distension. In our recent hydrodilatation approach [19] targeting the anterior and inferior glenohumeral capsules, a total of 30 mL was administered, with 15 mL per injection site. Although reported shoulder hydrodilatation volumes based on the meta-analysis by Wu et al [12] range from 10 to 90 mL, this cadaveric study aimed to determine the minimal volume required to achieve full joint infiltration. Excessive volumes could obscure the precise dye distribution by overwhelming the true spreading pattern.

### Clinical implications

These findings hold important clinical significance. The posterior glenohumeral injection consistently infiltrated the posterior glenohumeral joint but demonstrated limited diffusion to the anterior glenohumeral joint and the rotator interval. Given that fibrosis and contracture in adhesive capsulitis predominantly affect these anterior structures [20, 21], anteriorly directed approaches—such as the rotator interval or dual-target injections—are likely to yield superior therapeutic outcomes. Both techniques provide direct access to the rotator interval, a critical region implicated in restricted external and internal rotations of the shoulder. The dual-target injection offers additional advantages when adhesive capsulitis coexists with (or clinically mimics) subacromial impingement. Yet, it enables delivery of the injectate to both the glenohumeral joint and the subdeltoid bursa. Effective bursal infiltration, however, requires confirmation of the lateral spread by fenestrating the connecting portion of the subdeltoid bursa that attaches to the lateral acromial edge and the coracoacromial ligament. Additionally, our study demonstrated that the long head of the biceps tendon was not consistently stained in the posterior glenohumeral joint injection group. Given that the pathology of the long head of the biceps tendon is commonly associated with shoulder disorders, injections administered solely via the posterior glenohumeral approach may not adequately address concomitant biceps-related pathology. This observation provides further rationale for employing dual-target and rotator interval approaches when managing patients with adhesive capsulitis. In summary, the selection of injection technique should be guided by the predominant pathological target: anteriorly directed approaches for adhesive capsulitis, dual-target injections for combined capsulitis and bursopathy, and posterior injections for pathology confined to the posterior capsule.

### Study limitations

This cadaveric study has several limitations. First, the use of embalmed cadavers—although preserved using a Fix-for-Life method to maintain tissue pliability—may not fully replicate the biomechanical and fluid dynamic properties of living tissue. Second, the relatively small sample size (18 shoulders) limits the generalizability of our findings and may have reduced statistical power, thereby limiting the ability to detect subtle between-group differences. Accordingly, nonsignificant results should be interpreted with caution, as they may reflect insufficient power rather than a true absence of effect. Larger-scale studies are warranted to validate these findings and to further elucidate outcomes that did not reach statistical significance. Third, dye distribution served as a surrogate for injectate spread. Although green dye provided clear visualization, it may not accurately represent the diffusion behavior of therapeutic agents in vivo. Fourth, the cadavers in this study were not characterized with respect to the presence or absence of adhesive capsulitis. In addition, glenohumeral joint compliance after the Fix-for-Life embalming process may differ from that in living patients, potentially affecting injectate distribution. Future investigations using in vivo imaging modalities, such as contrast-enhanced computed tomography, magnetic resonance imaging or fluoroscopy, are warranted to determine whether the dye spread patterns observed in this cadaveric study can be replicated in clinical populations.

## Conclusions

This cadaveric study demonstrated that the injection technique markedly affects the injectate distribution within the shoulder. The rotator interval and dual-target approaches consistently infiltrated the biceps tendon sheath and the anterior glenohumeral joint, with the dual-target technique providing the most reliable bursal coverage. Conversely, the posterior glenohumeral approach achieved consistent posterior glenohumeral joint infiltration but limited anterior spread. These results indicate that the injection strategy should be tailored according to the primary pathology—anterior approaches for adhesive capsulitis, dual-target injections for concurrent bursopathy, and posterior injections for isolated posterior capsular disease.

## Supplementary information

ELECTRONIC SUPPLEMENTARY MATERIAL

## Data Availability

The datasets used and/or analyzed during the current study are available from the corresponding author upon reasonable request.
