# Supplementary material for: Cadaveric analyses of injectate distribution patterns in ultrasound-guided rotator interval, dual-target, and posterior glenohumeral injections
Source: Insights Imaging. 2026 Mar 18;17:77. doi: 10.1186/s13244-026-02255-y (PMC13000091; doi:10.1186/s13244-026-02255-y)
Supplement: Supplementary file 1 — ELECTRONIC SUPPLEMENTARY MATERIAL [file 13244_2026_2255_MOESM1_ESM.pdf]

Cadaveric Analyses of Injectate Distribution Patterns in Ultrasound-Guided Rotator Interval, Dual-Target, and Posterior Glenohumeral Injections

ELECTRONIC SUPPLEMENTARY MATERIAL

Insights Imaging (2026) Naňka O, Mezian K, Chang KV, Wu WT, Ricci V, Özçakar L.

Supplemental Figure 1. No staining observed, with dye *(black arrowheads)* confined to the connective tissue contiguous with the superficial portion of the lateral bundle of the coracohumeral ligament **(A)**, and partial infiltration *(black arrows)* within the infraspinatus muscle and fossa **(B)**.

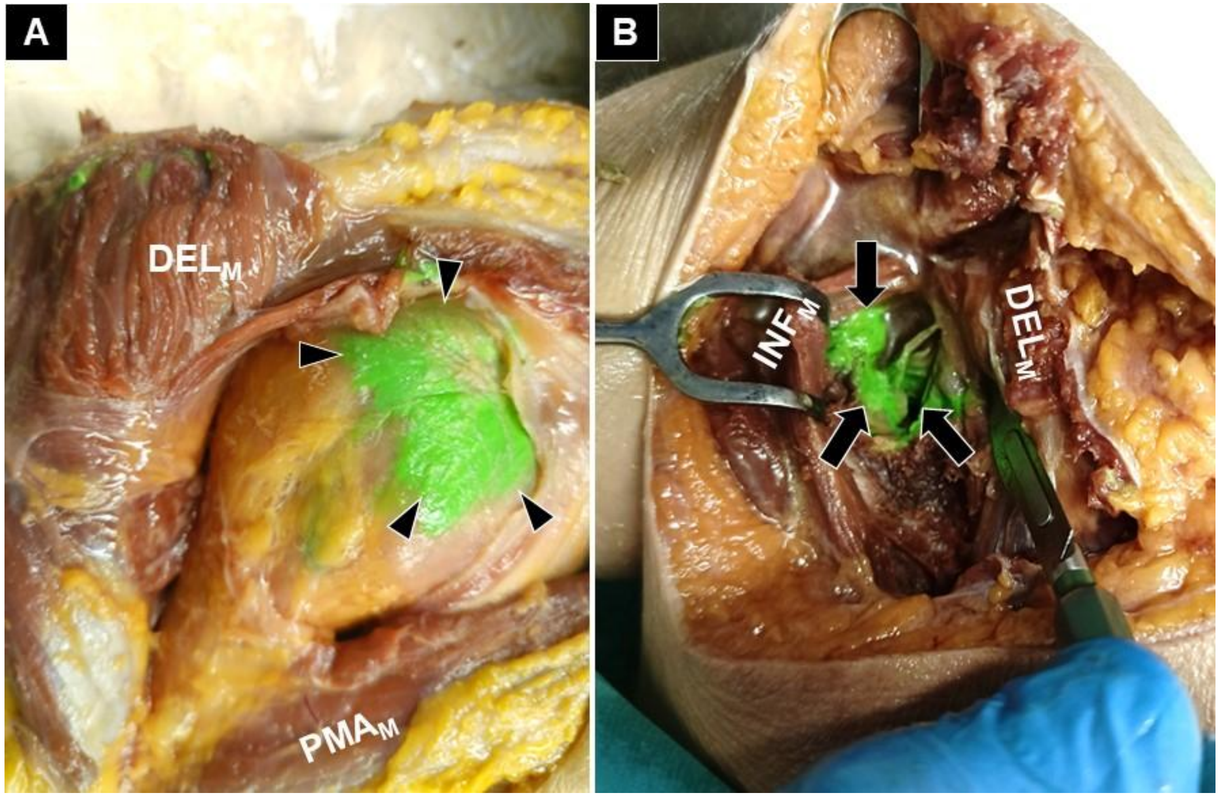

PMA$_M$, pectoralis major muscle; DEL$_M$: deltoid muscle; INF$_M$, infraspinatus muscle.
